# Mitochondrial Mutations in Cardiovascular Diseases: Preliminary Findings

**DOI:** 10.3390/genes15111442

**Published:** 2024-11-08

**Authors:** Anastasios Papageorgiou, Fragkiski-Ioanna Sofiou, Panagiotis Lembessis, Lubomir L. Traikov, Nina-Rafailia Karela, Dimitrios C. Angouras, Anastassios Philippou

**Affiliations:** 1 Department of Physiology, Medical School, National and Kapodistrian University of Athens, 11527 Athens, Greece; tasos1998p@hotmail.com (A.P.);; 2 Department of Medical Physics and Biophysics, Medical University, 1431 Sofia, Bulgarialltraikov@hotmail.com (L.L.T.); 3 Department of Cardiac Surgery, Attikon University Hospital, Medical School, National and Kapodistrian University of Athens, 11527 Athens, Greece

**Keywords:** coronary artery disease, mitochondrial DNA, *ND1* gene

## Abstract

**Background/Objectives**: Mitochondria are the main organelles for ATP synthesis able to produce energy for several different cellular activities. Cardiac cells require high amounts of energy and, thus, they contain a high number of mitochondria. Consequently, mitochondrial dysfunction in these cells is a crucial factor for the development of cardiovascular diseases. Mitochondria constitute central regulators of cellular metabolism and energy production, producing approximately 90% of the cells’ energy needs in the form of ATP via oxidative phosphorylation. The mitochondria have their own circular, double-stranded DNA encoding 37 genes. Any mitochondrial DNA sequence anomaly may result in defective oxidative phosphorylation and lead to cardiac dysfunction. **Methods**: In this study, we investigated the potential association between mitochondrial DNA mutation and cardiovascular disease. Cardiac tissue and serum samples were collected from seven patients undergoing coronary artery bypass grafting. Total DNA was extracted from cardiac muscle tissue specimens and serum and each sample was subjected to polymerase chain reaction (PCR) to amplify the NADH dehydrogenase 1 (*ND1*) gene, which is part of the mitochondrial complex I enzyme complex and was screened for mutations. **Results**: We identified one patient with a homoplasmic A to G substitution mutation in cardiac tissue DNA and two patients with heteroplasmic A3397G mutation in serum DNA. Specifically, amplicon sequence analysis revealed a homoplasmic A3397G substitution in the *ND1* gene in a tissue sample of the patient with ID number 1 and a heteroplasmic mutation in A3397G in serum samples of patients with ID numbers 3 and 6, respectively. The A to G substitution changes the amino acid from methionine (ATA) to valine (GTA) at position 31 of the *ND1* gene. **Conclusions**: The detection of this novel mutation in patients with coronary artery disease may contribute to our understanding of the association between mitochondrial dysfunction and the disease, implying that mitochondria may be key players in the pathogenesis of cardiovascular diseases.

## 1. Introduction

Mitochondria are the key organelles essential for energy production generating approximately 90% of the cells’ energy needs in the form of ATP via oxidative phosphorylation, carried out by the electron transport chain (ETC) protein complexes located in their inner membrane. Mitochondrial function is not restricted to ATP synthesis; they are involved in other essential cellular processes including apoptosis, mitophagy, and ion regulation [1,2]. Unlike nuclear DNA (nDNA), the mitochondrial DNA (mtDNA) is a circular, double-stranded structure which, in coordination with the nDNA, contributes to the assembly of the mitochondrial respiratory (electron transport) chain proteins. It consists of 16.6 Kbp, lacks intron–exon regions, and contains 37 genes, of which 13 encode for ETC polypeptides, 2 encode for the mitochondrial ribosomal RNA (mt-rRNAs) necessary for their synthesis, and 22 encode for tRNAs needed for the intra-mitochondrial synthesis of the 13 ETC proteins [3]. Replication, transcription, and translation of mtDNA are all controlled by a single highly mutable non-coding region, the displacement loop (D loop) [4]. The D-loop region is 6–17 times more mutable than nuclear DNA and constitutes the most polymorphic region of the mitochondrial genome [5], primarily due to the absolute absence of histones in its structure, rendering it susceptible to reactive oxygen species (ROS), ultraviolet (UV), and other agents that may cause damage within cells.

The mitochondrial copy number within a cell may vary according to cell and tissue type, ranging from 2 to 100 mitochondria per cell, and each mitochondrion is estimated to contain 2–10 copies of mtDNA [6,7,8,9,10]. Mitochondria are dynamic organelles. Their morphology may vary, and they may undergo fusion or fission with a cell depending on the needs of the cell [1]. Mitochondrial DNA alterations, depending on the degree of heteroplasmy, may contribute to several human diseases including cancer, neurodegeneration, diabetes mellitus, encephalopathy, muscle weakness, renal dysfunction, and cardiomyopathy [11,12]. Recent studies suggest that mitochondrial dysfunction is associated with numerous cardiovascular and metabolic disorders, such as coronary artery disease (CAD), hypertension, diabetes mellitus, obesity, cardiac hypertrophy, heart failure (HF), and ischemia–reperfusion (I/R) injury [13,14,15]. Specifically, mitochondrial dysfunction and decreased ATP production result in disease development through two distinct pathophysiological pathways, i.e., inadequate energy supply and increased ROS production [12].

Also, mtDNA is unstable in somatic cells and, therefore, de novo mutations may accumulate and lead to mitochondrial and tissue dysfunction. Mitochondrial dysfunction leads to changes in mitochondrial biogenesis and metabolic processes, increased production of ROS, and mitochondrial damage. Consequently, such changes in cardiac cells and tissue can affect the normal function of the myocardium [16]. Furthermore, variation in the mtDNA sequence is common in certain tumors and neurodegenerative disorders. Mitochondrial mutations are common in cancer cells, where they confer a survival advantage and give the energy that tumor cells need [12,17].

There are several risk factors that can change the normal function of mitochondria. These include vasoactive agents that lead to oxidative stress, atherosclerosis, and hypertension [8,18]. Large- and medium-sized arteries are affected by atherosclerosis, causing several cardiovascular diseases. Monocytes and macrophages have been detected in patients with increased low-density lipoprotein (LDL) and cholesterol levels on artery walls, leading to problems in circulation and increasing the risk for thrombosis [18]. Moreover, smoking is related to increased oxidative stress and ROS production. ROS lead to severe changes that interfere with normal endothelial cell function [19]. They result in an increased inflammatory response and production of NADPH oxidases and, along with several inflammatory agents such as cytokines/chemokines, they contribute to atherosclerotic plaques [20].

The core subunit of the mitochondrial membrane respiratory chain NADH dehydrogenase (complex I) catalyzes the electron transfer from NADH through the respiratory chain, using ubiquinone as an electron acceptor. The NADH-ubiquinone oxidoreductase core subunit 1 (MT-ND1) in the mitochondrial genome is responsible for encoding the corresponding protein. The NADH dehydrogenase 1 (ND1) subunit, among several other subunits of complex I, is found within the hydrophobic membrane region as part of the core proton translocation machinery of the complex. In addition to its respiratory function, the ND1 subunit also serves an important structural function, forming part of the ubiquinone binding pocket at the interface between the membrane and matrix regions [21]. Its associated biological processes include respiratory electron transport, ATP synthesis by chemiosmotic coupling, and heat production [10,22].

Both animal and human studies support the hypothesis that the *ND1* gene is related to several different cardiovascular diseases including myocardial ischemia, cardiomyopathies, heart failure, and cardiac arrhythmias. In this study, we collected preliminary data from our limited human samples available to evaluate the potential relationship between the *ND1* gene and myocardial ischemia. The genetic portion that was used in our study is highly fragile and susceptible to several mutations such as deletions, substitutions, and transition mutations. Specifically, we screened for mutations in the *ND1* gene in the myocardial tissue of patients undergoing coronary artery bypass grafting (CABG) surgery. We hypothesized that there may be associations between cardiovascular disease and *ND1* gene mutations. Thus, the aim of the study was to determine the potential relationship between CAD and *ND1* mutations-induced mitochondrial disorders, and to evaluate how the presence of mtDNA mutations affect cardiovascular function [20]. For this purpose, we collected blood as well as cardiac and vascular tissue samples from the right atrium and ascending aorta, respectively, of those patients. Risk factors, comorbidities, the extent of CAD, and left ventricular ejection fraction were also recorded to allow for an approximate quantitative association of potential mtDNA aberrations with atherosclerotic risk factors, coronary atherosclerotic burden, and the resultant myocardial damage (Table 1).

## 2. Materials and Methods

In the present study, we clinically examined the data from seven patients with coronary artery diseases admitted to the cardiology department to perform necessary cardiac surgery. The target population included patients with the presence of CAD, in combination with several associated risk factors. The main risk factors that we detected in most patients were arterial hypertension, in combination with smoking. Five out of seven patients had dyslipidemia/hyperlipidemia, three had diabetes mellitus (DM), and one patient suffered from obesity. Interestingly, two of the patients, the patient with ID number 4 and the patient with ID number 5, had cataract surgery; studies have shown that cataract patients have a higher risk of developing CAD compared to the rest of the population [23]. Aortic and right atrial myocardial tissue samples were collected intraoperatively from these patients. In addition, patient with ID number 3 (AKTis) underwent concomitant aortic valve replacement (AVR) for severe symptomatic aortic stenosis. Tissue samples were placed in isotonic electrolyte solution (Kreb’s–Ringer’s bicarbonate buffer, K4002, Sigma Aldrich, St. Louis, MO, USA) and stored at −80 °C till use. Blood serum from each patient was also collected and stored at −20 °C. Blood samples were allowed to clot at room temperature in EDTA-free tubes for 30 min and serum was collected following centrifugation at 2000× *g* at 4 °C for 10 min.

Established protocols with minor modifications for DNA extraction from human serum and cardiac tissue were applied [11,24]. Briefly, frozen cardiac tissue was thawed to room temperature, weighed, and rinsed with PBS. Tissues were then placed in tissue lysis buffer (100 mM Tris-Cl, 200 mM NaCl, 0.2% *w*/*v* SDS, 5 mM EDTA and 100 μg/mL proteinase K) and lysed by proteinase K incubation at 56 °C for 3 h. This was followed by purification via phenol–chloroform extraction and ethanol precipitation. Similarly, 250 μL of serum samples was mixed with 250 μL of PBS and subjected to proteinase K digestion, followed by phenol–chloroform extraction and ethanol precipitation. The quantity and purity of each extracted DNA were verified by spectrophotometric analysis (Shimadzu BioSpec-nano, Shimadzu Corporation, Kyoto, Japan) and 1% agarose gel electrophoresis.

### 2.1. Polymerase Chain Reaction

The tissue DNA was dissolved in 50 μL of molecular biology-grade nuclease-free H_2_O, while the serum DNA was dissolved in 30 μL of nuclease-free molecular biology-grade H_2_O (Takara Bio Inc., Otsu, Shiga, Japan). The concentrations of each extracted DNA are in Table 2.

**Figure 1:**
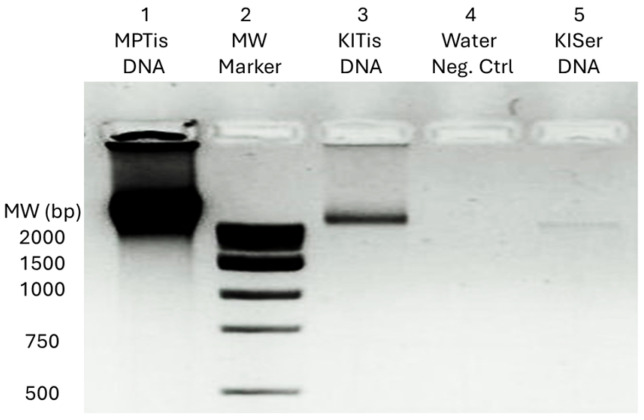
DNA extraction for molecular genetic analyses of mtDNA mutations. Lanes 1 and 3 show DNA extracted from tissue (Tis). Lane 2 shows the molecular weight (MW) marker. Lane 4 shows extraction using water instead of samples (Neg Ctrl). Lane 5 shows DNA extracted from serum (KlSer DNA). MP and KI refer to patient identification and Tis refers to “Tissue” specimen type. Similarly, “KI” refers to patient identification and “Ser” refers to “Serum” specimen type. It is noted that the faint serum band reflects the low amount of DNA isolated from the serum samples [cell-free DNA (cfDNA) present in the circulating serum].

**Figure 2:**
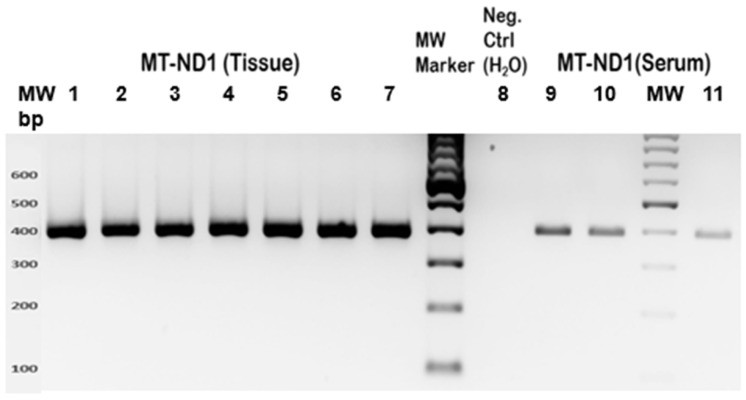
PCR amplification of tissue and serum DNA in the *ND1* gene region. Amplicons of tissue samples (Lanes 1–7) and serum samples (Lanes 9, 10, 11) are shown. Lane 8 represents the negative control where DNA was replaced with nuclease-free H_2_O. The samples represent the patients shown in Table 1 as follows: sample 1—ID 6; sample 2—ID 2; sample 3—ID 5; sample 4—ID 7; sample 5—ID 4; sample 6—ID 1; sample 7—ID 3; sample 9—ID 6; sample 10—ID 3; sample 11—ID 7; M: MW marker. It is noted that while there are detectable levels of circulating DNA yielded from serum, there is considerably less signal in these samples, because there is much less DNA in serum compared with tissue.

**Figure 3:**
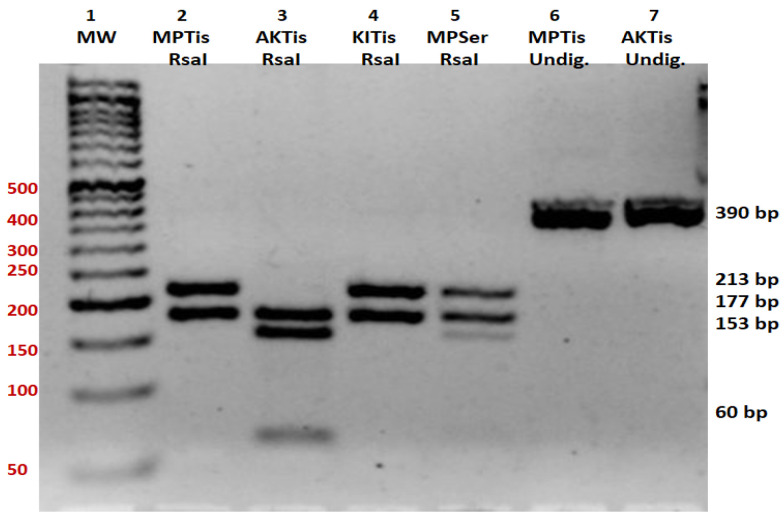
Restriction fragment length polymorphism (RFLP) analysis of the A3397 substitution mutation. An RsaI restriction site is created by the A to G transition at position nt 3397 of the ND1 subunit. Lane 1: molecular weight marker (MW); Lane 2: RsaI restriction digestion of sample MPTis; Lane 3: RsaI restriction digestion of sample AKTis; Lane 4: RsaI restriction digestion of sample KITis; Lane 5: RsaI restriction digestion of sample MPSer; Lane 6: MPTis; Undig.: undigested amplicon of sample MPTis; Lane 7: AKTis; Undig.: undigested amplicon of sample AKTis.

**Figure 4:**
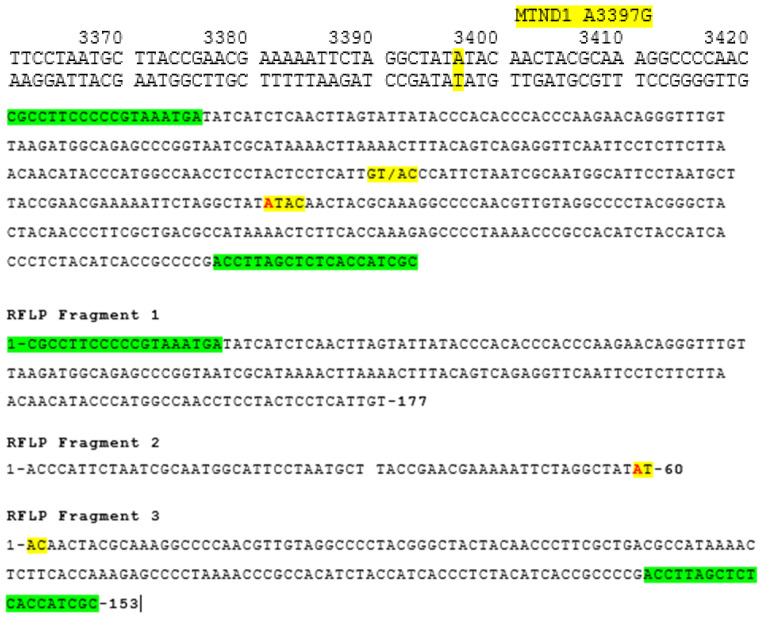
Normal genetic sequence of the *ND1* gene. Normal Sequence: 177 + 213; A to G transition: 177+ 60 + 153. RsaI will cut at two sites: 123 ACTCCTCATT GT
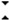
AC CCATTCTAAT and at 183 TCTAGGCTAT GT/AC AA
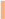
CTACGCAA to yield 134 and 194 bp fragments. The green highlights are the position of the primers used to amplify the region. The yellow fonts indicate the restriction site of RsaI. The red font indicates the A transition to G that creates a second restriction site for RsaI.

**Figure 5:**
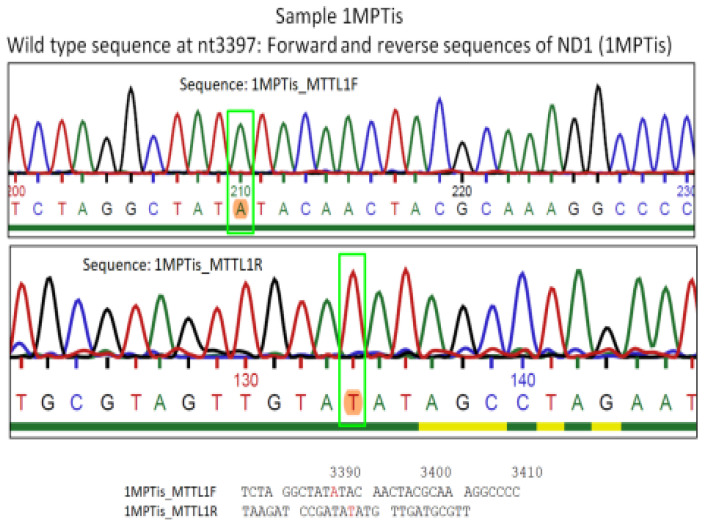

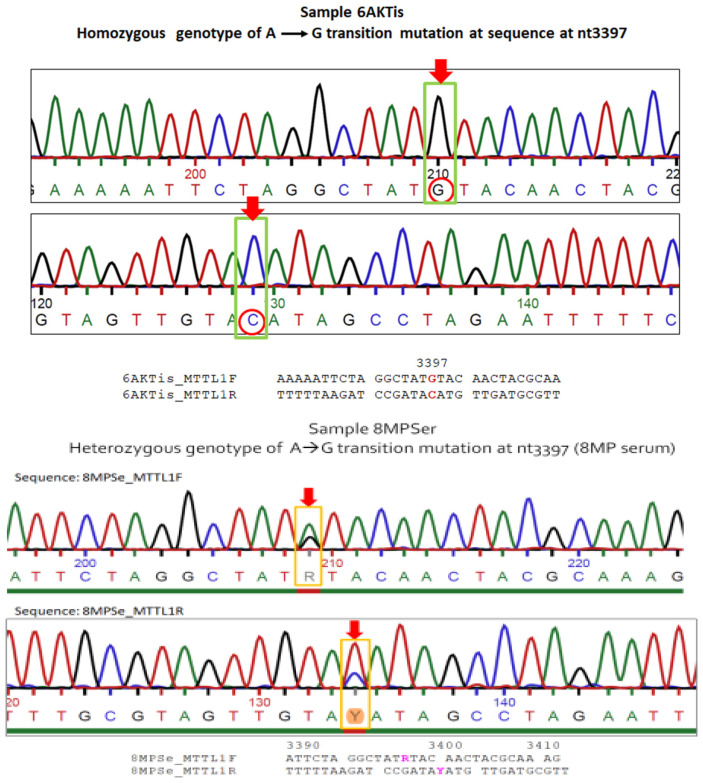
Chromatogram of the DNA sequencing analysis of the *MT*-*ND1* gene. The amplicons were subjected to sequence analysis of the forward and reverse strands of the *ND1* gene. Upper panel: normal genetic sequence of the *ND1* gene in sample MPTis. Middle panel: mutated genetic sequence of the *ND1* gene in sample AKTis. The sequence analysis revealed an A to G substitution of homoplasmic genotype. Lower panel: Sequence analysis of the *ND1* gene in sample 8MPSer; A to G substitution of heteroplasmic genotype: R = A + G, Y = C + T (see also Figure 6). The arrow n in the colored boxes emphasize the mutation location of the amplicon.

Primer sets were designed using Primer3 (v. 0.4.0) software to amplify a 389 bp region of the *ND1* gene (Figure 1 and Figure 2): Forward Primer Sequence: 5′-GCGCCTTCCCCCGTAAATGA-3′; Reverse Primer Sequence: 5′-GCGATGGTGAGAGCTAAGGT-3′. The size of the amplicon was confirmed by restriction fragment length polymorphism (RFLP), as shown in Figure 3 (see also Figure 4), and by direct sequencing, as shown in Figure 5. A 20 μL PCR was performed using the Kapa SYBR Fast Universal (Takara Bio KK4607, Takara Bio Inc., Otsu, Shiga, Japan) with 0.3 μM primer concentration. PCR amplification was performed on 0.25 μg of tissue DNA and 2 μL of serum DNA on a Verity thermal cycler (Applied Biosystems, Foster City, CA, USA) under the following cycling conditions: one cycle of denaturation at 95 °C for 3 min, forty cycles of amplification at 95 °C for 30 s, 57 °C for 30 s, and 72 °C for 30 s. This was followed by one cycle at 72 °C for 3 min (Figure 2). Then, the amplicons were subjected to restriction fragment length polymorphism and sequence analysis. Primers were designed to amplify a 389 bp region of the *ND1* gene. The size of the amplicon is 389 base pairs. This was confirmed by restriction fragment length polymorphism (RFLP), as shown in Figure 3, and by direct sequencing, as shown in Figure 5. The starting DNA material yielded by the serum extraction was less than that of the tissue samples and therefore, the endpoint PCR amplification product was much less. However, the quantity yielded was sufficient for sequencing to proceed.

### 2.2. Restriction Fragment Length Polymorphism (RFLP)

Restriction fragment length polymorphism of the *ND1* amplicon with RsaI was carried out and the products were electrophoresed on a 2.5% agarose gel to determine the size of the digests (Figure 3). The normal sequence of the *ND1* gene has an RsaI restriction site at nt 3338 (NC_012920, gi:251831106 Revised Cambridge Reference Sequence (rCRS) of the Human mtDNA) which yields a 177 and a 213 bp band (Figure 3). The A to G substitution at nt 3397 of the ND1 subunit introduces an additional restriction site, yielding three bands of 177 bp, 60 bp, and 153 bp. We identified an A to G substitution in codon 31 of the *ND1* gene, which converts the amino acid Met to Val. The A to G mutant at nucleotide (nt) 3397 has created an additional restriction site at 3397, yielding three bands of 177, 60, and 153 bp [25]. Specifically, the restriction endonuclease enzyme RsaI recognizes the 5′-GT/AC-3′ sequence in the 391 bp mitochondrial sequence amplified by specific primers. The substitution of A by G at nucleotide 237 has created an additional restriction from ATAC to GTAC. Thus, a homoplasmic A to G substitution mutation was detected in cardiac tissue of one patient with ID number 1 (6AKTi) and a heteroplasmic mutation in the sera of two other patients with ID numbers 3 and 6 (Figure 3 and Figure 4).

### 2.3. DNA Sequence Analysis

DNA sequencing is the process of determining the exact sequence of nucleotides (bases) in DNA and includes any method or technology that is used to determine the order of the four bases: adenine, guanine, cytosine, and thymine. In this study, the amplicons were sequenced and analyzed using the Chromas v. 2.6.6 software for DNA sequencing and analysis (Technelysium Pty Ltd., South Brisbane QLD 4101, Australia). We further performed amplicon sequence alignment using the Clustal Omega (1.2.4) multiple sequence alignment software [26].

## 3. Results

**Figure 6:**
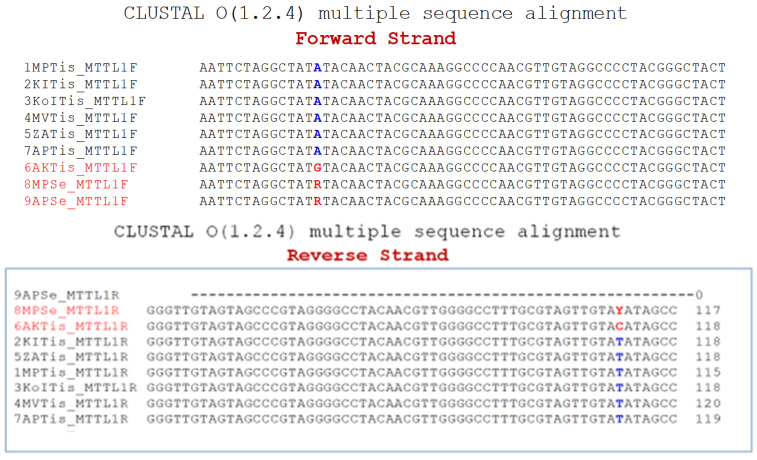
Sequence analysis of the *ND1* gene. Multiple sequence alignment of MT-ND1 (forward strand—F; reverse strand—R) of the samples using the CLUSTAL O (1.2.4) multiple sequence alignment software. The presence of a homoplasmic A to G mutation in sample 6AKTis and a heteroplasmic A/G mutation in samples 8MPSe and 9APSe is highlighted in red characters. More specifically, the tissue sample of patient 6AKTis shows a homoplasmic A to G substitution mutation, while serum samples 8MPSe and 9APSe show heteroplasmy for the A/G mutation (R = A + G; Y = C + T). The sequence of the remaining six samples (1MPTis, 2KITis, 3KoITis, 4MVTis, 5ZATis, and 7APTis) shows a normal genotype. The blue and red colors of the letters are intended to highlight the normal sequence and the mutation, respectively.

Sequence analysis of the forward and reverse strands of the *ND1* gene in sample 6AKTis revealed an A to G substitution (Figure 5 and Figure 6). Also, a heterozygous A to G substitution mutation was detected in patient MPSer. The normal sequence of the *ND1* gene has an RsaI restriction site at nt 3338, which yields a 177 bp band and a 213 bp band (Figure 3). We identified an A to G substitution at nucleotide 3397, which converts the highly conserved amino acid methionine (ATA) to valine (GTA) at codon 31 of the *ND1* gene [NC_012920 gi:251831106 Revised Cambridge Reference Sequence (rCRS) of the Human 212 Mitochondrial DNA]. Restriction digestion of the 391 bp fragment of the amplicon sequence of patient samples 1MPTis and 2KITis yielded two fragments of 178 bp and 213 bp in size, as determined by electrophoresis on a 2.5% agarose gel, indicating a normal sequence (Lanes 2 and 4, Figure 3), while that of patient sample AKTis yielded three fragments of 178 pb, 153 bp, and 60 bp [25], indicating the presence of a new restriction site for RsaI created by the A to G substitution (Lane 3, Figure 3). Additionally, patient sample MPSer is heteroplasmic for the A to G substitution as indicated by the four fragments produced by RsaI digestion (Lane 5, Figure 3). The undigested amplicons for patient samples MPTis and AKT are shown in Lanes 6 and 7 (Figure 3). Specifically, a homoplasmic A to G substitution mutation was detected in the cardiac tissue of one patient (with the ID number 1, Table 1) and a heteroplasmic mutation in the sera of two other patients (with the ID numbers 3 and 6, respectively, Table 1) (Figure 3 and Figure 4). The serum samples analyzed in the present study, which had no differences or mutations compared to the normal mitochondrial genome, were not included in the representative normal sequence analyses (Figure 6).

## 4. Discussion

The present study identified the A3397G substitution mutation of the core ND1 subunit of the mitochondrial membrane respiratory chain NADH dehydrogenase (complex I) in three patients undergoing coronary artery bypass grafting. There is sufficient evidence indicating that mtDNA mutations contribute to the phenotype in patients with coronary artery diseases, such as hypertension-related mutations, ischemic heart diseases, and Brugada syndrome, which is characterized by a structurally normal heart but with typical electrocardiogram alterations and a high risk of sudden death. Different studies have shown that mutations mainly affect the mtDNA copy number, energy production, protein synthesis, overproduction of mitochondrial reactive oxygen species, and Ca^2+^ metabolism [27,28].

Specifically, substitutions in the mitochondrial *ND1* gene have been associated with several different diseases including mitochondrial encephalomyopathy, lactic acidosis and stroke-like episodes (MELAS), cardiomyopathy and diabetes mellitus, LHON, Wolfram syndrome, and maternal inherited diabetes, suggesting that changes in this DNA codon of the *ND1* gene may be associated with very diverse pathogenic processes [21,25,29].

The A3397G substitution is a rare mutation that has been associated with Alzheimer’s and mitochondrial myopathies [21,29,30,31,32,33,34]. This substitution mutation changes a highly conserved methionine (ATA) to valine (GTA) at amino acid 31 and has previously been found in Alzheimer’s disease and Parkinson’s disease patients [25], potentially indicating a pathogenic mutation that compromises mitochondrial function. Moreover, substitutions of the highly conserved Met31 in the *ND1* gene have been found to be caused by the rare mitochondrial single-nucleotide polymorphisms (mtSNPs) A3397G and T3398C, which were previously identified in two patients with left ventricular noncompaction (LVNC) [35,36]. In that study, it was further reported that the A3397G mutation, among others, may lead to several mitochondrial myopathies and that this substitution mutation of the complex I ND1 subunit may disrupt mitochondrial function, resulting in LVNC. The heart is an organ highly dependent on oxidative metabolism, and defects in complex I are a potential contributor to cardiomyopathies. Whether the A3397G mutation causes these conditions alone or in combination with other mutations remains to be clarified [27].

Previous studies have shown that mutations in the *ND1* gene are strongly related to the formation of atherosclerotic plaques, resulting in the obstruction of coronary circulation with common symptoms, such as severe chest pain, physical inability, and sudden death [37]. Atherosclerosis is a multifactorial disease that is influenced by smoking, bad diet, diabetes mellitus, dyslipidemia, and genetic predisposition, as in the case of familial hypercholesterolemia. Interestingly, though, studies support the hypothesis that mitochondrial mutations play a significant role in the promotion of more severe atherosclerotic changes [35,38].

In this study, the patients identified with the *ND1* gene mutation had a similar clinical phenotype, with severe multiple-vessel atherosclerotic lesions and cardiovascular dysfunction caused by myocardial ischemia and infarction. In addition, arterial hypertension, accompanied by more atherosclerotic lesions in coronary vessels as well as in the common iliac artery, was recorded in one of these patients. Considering the multi-systemic and multi-symptomatic nature of the ND1 subunit anomalies, our findings suggest that the presence of the A3397G genotype in our subjects is associated with their disease phenotype. Nevertheless, it may be difficult to determine the contribution of mtDNA mutations, including that of A3397G, since the phenotypic expression and the penetrance depend on the affected tissue where the mutant phenotype develops and when a certain critical threshold of mutation heteroplasmy is reached. Thus, the mechanism of the A3397G genotype’s contribution to disease penetrance needs further investigation. Mitochondrial mutations result in alteration of the energy status in skeletal and cardiac muscle cells, leading to decreased energy storage in these cells [29]. Especially for the cardiac muscle tissue, mitochondrial mutation of the *ND1* gene is the leading cause of severe oxidative stress reactions and production of ROS, which result in the instability of atherosclerotic plaques, making them more vulnerable. This may lead to endothelial rupture, consequent clot formation, and further, partial or total obstruction of the blood vessels, as in coronary artery disease [35,38].

The detection of mtDNA mutations may help predict the probability of developing certain cardiovascular diseases in the general population. The creation of an mtDNA database with different disease conditions may provide a better genotype–phenotype grouping of patients with myocardiopathies. Also, new technologies such as next-generation sequencing (NGS) and bioinformatics may provide accurate, quick, and affordable screening tools. Earlier diagnosis can encourage mutation carriers to attend more frequent follow-ups and adopt preventive practices, such as lifestyle changes (e.g., diet, smoking cessation), and prevent the disease with specific medications. The detection and analysis of circulating mtDNA may serve as a potential non-invasive diagnostic and prognostic biomarker in heteroplasmic shifts of mtDNA and disease monitoring.

Nevertheless, it must be considered that mitochondrial diseases may be associated with mutations in nDNA. Considering that mitochondrial disorders are multi-systemic, the clinical significance of nDNA and mtDNA mutations in cardiomyopathies needs further investigation and analysis. Interactions between mitochondrial and nuclear gene products contribute to mitochondrial ATP production, mitophagy, and redox regulation, and any anomalies in either mtDNA or nDNA may affect these processes. Thus, a thorough understanding of the pleiotropic nature of mitochondrial energy production may be necessary to understand disease phenotypes.

The advent of NGS technologies in recent years, such as whole-genome, whole-exome, whole-mtDNA, targeted-exome, and RNA sequencing, may improve the detection and analysis of gene expression and the effects of point mutations and deletions in genes involved in mitochondrial function [36,39]. Additionally, NGS methods can offer a precise diagnosis of the mitochondrial mutations and, thus, provide a means of monitoring mitochondrial heteroplasmic shifts to reduce mutation load research and increase clinical applications. This study provides evidence for the presence of a mitochondrial mutation in cardiac tissue and serum derived from human subjects undergoing cardiac surgery and suggests its contribution to mitochondrial disorders in the pathophysiology of ischemic heart disease. The contributions of the *ND1* A3397G mutation and its protein product to ischemic heart disease need to be further investigated to confirm whether this mitochondrial mutation could be used as a potential prognostic biomarker in cardiovascular diseases. A main limitation of our study is the small sample size; thus, further investigation with a larger sample size is required to buttress the contribution of the A3397G to CVD and reveal whether a possible synergy of A3397G with other mutations contributes to the phenotypic penetrance of CAD. In addition, data at the protein level that could bolster our arguments by potentially lining up any protein anomalies with each patient’s *ND1* DNA status are missing in this study due to the small (tissue) volume and the depletion of the samples during the standardization process of the analyses. Another limitation of this study is that the patients exhibited diverse clinical characteristics. Their comorbidities were variable; three had mitral and tricuspid valve insufficiency and two of them were suffering from renal dysfunction. Thus, the preliminary results of the study should be interpreted with caution.

## 5. Conclusions

This study identified a substitution mutation A3397G of the core *ND1* gene in the cardiac tissues and serum of patients undergoing cardiac surgery. The first patient was identified as homoplasmic in this mutation (A to G) and the second as heteroplasmic (A to R, which includes A and G bases). Both patients have similar clinical profiles with severe multiple-vessel atherosclerotic lesions and cardiovascular dysfunction caused by myocardial ischemia and infarction, dyslipidemia, and obesity. The presence of the *ND1* A3397G mutation in patients with coronary artery disease may contribute to our understanding of the possible association between mitochondrial dysfunction and disease phenotype. 

## Figures and Tables

**Table 1:** Clinical characteristics of the patients who participated in this study.

ID	Age	Risk Factors	Diseases	Comorbidities	Type of Surgery	Symptoms
1	59	HTN Obesity Dyslipidemia Smoking	Double-vessel CAD Angina	Mild insufficiency of mitral and tricuspid valve, peripheral arteriopathy, previous duodenal ulcer	Double-vessel bypass surgery	-
2	62	Dyslipidemia Smoking	Triple-vessel CAD STEMI	Previous duodenal ulcer	Four-vessel bypass surgery	Ventricular tachycardia
3	71	HTN DM Hyperlipidemia Smoking	Severe aortic stenosis, double-vessel CAD- NSTEMI	Mild mitral, tricuspid insufficiency, mild renal dysfunction, carotid artery stenosis left and right	Double-vessel bypass surgery, replacement of aortic valve	Angina, dyspnea, pulmonary edema, systolic murmur of aortic valve
4	81	HTN Dyslipidemia Smoking	Triple-vessel CAD NSTEMI	Aneurysm of abdominal aorta, renal dysfunction, pancreatitis, cataract	Four-vessel bypass surgery	-
5	66	HTN DM	Triple-vessel CAD-NSTEMI Angina	Cataract surgery	Triple-vessel coronary artery disease	-
6	64	Obesity HTN Dyslipidemia Smoking	Double-vessel CAD-NSTEMI	Mild renal dysfunction, mild insufficiency of mitral and tricuspid valve	Triple-vessel bypass surgery	-
7	71	HTN DM, past smoking, family history	Triple-vessel CAD-NSTEMI	-	Four-vessel bypass surgery	Angina pain

HTN: hypertension; DM: diabetes mellitus; CAD: coronary artery disease; STEMI: ST-elevation myocardial infarction; NSTEMI: no-ST-elevation myocardial infarction.

**Table 2:** Concentration of each DNA sample extracted from tissue and serum samples of the patients.

ID Number	Sample ID	DNA Conc (ng/mL)
1	1MP_Tis	109.85
	1MP_Ser	5.32
2	2KaI_Tis	221.9
	2KαI_Ser	8.69
3	3KozΙ_Tis	43.12
	3KozI_Ser	2.43
4	4MV_Tis	137.2
	4MV_Ser	2.50
5	5ZA_Tis	86.8
	5ZA_Ser	4.82
6	6AK_Tis	115.93
	6AK_Ser	2.76
7	7AP_Tis	45.77
	7AP_Ser	2.65

## Data Availability

The original contributions presented in the study are included in the article, further inquiries can be directed to the corresponding author.
